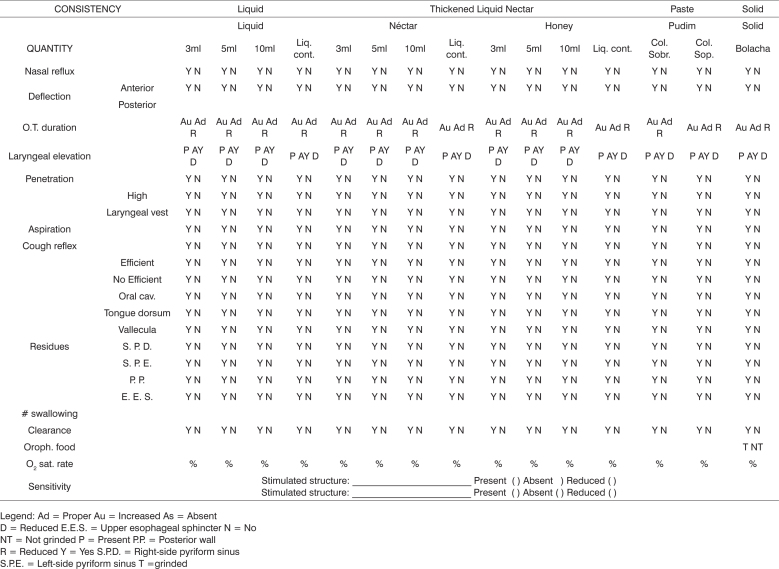# Interdisciplinary evaluation of dysphagia: clinical swallowing evaluation and videoendoscopy of swallowing

**DOI:** 10.1016/S1808-8694(15)30537-1

**Published:** 2015-10-19

**Authors:** Marina de Sordi, Lucia Figueiredo Mourão, Ariovaldo Armando da Silva, Luciana Claudia Leite Flosi

**Affiliations:** 1 MSc student in health, interdisciplinarity and rehabilitation, speech therapist - ENT Ward - Disfagia HC-Unicamp; 2 PhD in Sciences - Unifesp, Professor of Speech Therapy - FCM Unicamp; 3 PhD in Medicine - ENT and Speech Specialist; Professor of Medicine - FCM Unicamp; 4 PhD student in Neurolinguistics. Clinical speech therapist

**Keywords:** swallowing disorder, neurodegenerative disorders, swallowing assessment

## Abstract

Patients with dysphagia have impairments in many aspects, and an interdisciplinary approach is fundamental to define diagnosis and treatment. A joint approach in the clinical and videoendoscopy evaluation is paramount.

**Aim:**

To study the correlation between the clinical assessment (ACD) and the videoendoscopic (VED) assessment of swallowing by classifying the degree of severity and the qualitative/descriptive analyses of the procedures.

**Study design:**

cross-sectional, descriptive and comparative.

**Materials and methods:**

held from March to December of 2006, at the Otolaryngology/Dysphagia ward of a hospital in the country side of São Paulo. 30 dysphagic patients with different disorders were assessed by ACD and VED. The data was classified by means of severity scales and qualitative/descriptive analysis.

**Results:**

the correlation between severity ACD and VED scales pointed to a statistically significant low agreement (KAPA = 0.4) (p=0,006). The correlation between the qualitative/descriptive analysis pointed to an excellent and statistically significant agreement (KAPA=0.962) (p<0.001) concerning the entire sample.

**Conclusion:**

the low agreement between the severity scales point to a need to perform both procedures, reinforcing VED as a doable procedure. The descriptive qualitative analysis pointed to an excellent agreement, and such data reinforces our need to understand swallowing as a process.

## INTRODUCTION

Multidisciplinary work in dysphagia is a common denominator advocated by researchers and clinicians, since dysphagic patients have losses in the medical, nutritional, physiotherapeutic, physiological and speech arenas, thus needing numerous professionals to serve all of their health care demands^1^, ^2^, ^3^. The health care team which works with dysphagia must include professionals from different fields (nurses, speech therapists, nutritionists, physical therapists, psychologists) and physicians of different specialties (ENTs, surgeons, neurologists, gastroenterologists, geriatricians, radiologists and others). The multidisciplinary evaluation of dysphagic patients is fundamental in determining the diagnosis and planning treatment.

The most known methods used to assess swallowing are the clinical evaluation of swallowing (CES) and the instrumental tests of video-fluoroendoscopy (VFE) and swallowing video-endoscopy (SVE). CES can not make a definitive diagnosis of dysphagia; however, it is a component which allows us to understand its nature^4^. Silva^5^ states that CES helps obtain information on location, characteristics, whether structural or functional, the underlying etiology, and it also establishes the effectiveness of some approaches. CES is interpretative and it is based on observing the oral phase.

Among the instrumental tests, VFE has been considered the “gold standard”; however, because of its high cost and scarcity of places where it can be performed, SVE has proven accessible and doable. Swallowing videoendoscopy is a simple test, of low cost and little invasiveness, besides being easily transportable, making it possible to do sequential evaluations in patients with mobility challenges. It allows one to observe the pharyngeal phase of swallowing; it allows the physician to order the patient to perform airway protection maneuvers - so as to help the physician guide the patient regarding a proper diet for these patients^6^. SVE provides structural and sensorial information on the Pharyngo-laryngeal region, letting the physician see the functional pharyngeal phase of swallowing, as well as the visualization of silent aspiration7. Studies describe the advantages and contributions of SVE for the functional assessment of swallowing and highlight the importance this test gains in the diagnosis of dysphagia^7^, ^8^, ^9^, ^10^.

Considering the importance of SVE in the diagnosis of dysphagia, otorhinolaryngologists gain relevance in the work team, being the professional responsible for performing the exam. The ENT is in charge of interpreting the SVE in its functional and anatomical aspects, and such data is fundamental for diagnosis. The speech therapist can work together with the ENT during the exam, suggesting the assessment of therapeutic strategies.

Considering the different functions of each swallowing assessment procedure it is necessary to understand and interpret the different signs and symptoms observed in order to pinpoint the participation of each evaluation procedure and thus establish the approach in cases of dysphagia.

## OBJECTIVE

To study the correlation between the clinical evaluation (CES) and swallowing video-endoscopy (SVE) by classifying the degree of severity and the qualitative/descriptive analysis of the two evaluation procedures.

## MATERIALS AND METHODS

The present cross-sectional, descriptive and comparative study was approved by the Ethics Committee of the institution under protocol # 796/2005. All the subjects signed the informed consent form.

The evaluations were carried out in the dysphagia ward - ENT of a hospital in the country-side of the state of São Paulo between March and December of 2006. This ward is geared specially to patients with neurogenic dysphagia, diagnosed with Parkinson's disease (PD), Amyotrophic Lateral Sclerosis (ALS) and Machado-Joseph Disease (MJD).

All the patients were submitted to CES and SVE. After the procedures, the results were discussed with the team, made up by an ENT physician, ENT residents, speech therapists and nutritionists.

CES and SVE followed the procedures proposed by the present study (Attachment 1), which was built from the protocols of other authors^4^, ^5^, ^8^, 10, 11, 12, 13 and that from Flosi-Santos [5][1]. For direct, clinical and endoscopic evaluation we used cold lemon juice in powder, dyed with green aniline in the liquid, honey (thick liquid) and pudim (paste) and solid (¼ cornstarch cookie), provided in the order aforementioned. The honey consistency was obtained by adding one table spoon of thickener (Thicken-easy®) to 100ml of water; the pudim consistency was obtained by adding 2 table spoons of the same product to 100 ml of water. The nomenclature used for the consistencies follows the standards from the American Dietetic Association14.

The CES was done in a direct and indirect manner. The indirect one includes interview, structural and sensitive evaluation of the oral cavity and administration of food. The neck was ausculted during rest, during saliva swallowing and before, during and after the swallowing of food. Later on, compensatory maneuvers were studied in order to achieve a safe swallowing.

SVE followed the procedures proposed (Attachment 1) and was carried out by an otorhinolaryngologist using the conventional video-endoscopic equipment. The speech therapist participated offering the different food quantities and consistencies to be studied, and also suggesting the evaluation of certain airway protection maneuvers. The exam was recorded in a DVD.

### Analysis methodology

The data was analyzed in the following fashion:

- Step 1 - CES and SVE analysis by means of classification according to severity scales.

The severity scale employed for CES was proposed by Furkim and Silva^15^, while the SVE findings were classified according to the scale proposed by Macedo et al.^16^. The criteria used by the scales are shown in the Attachment 1.

- Step 2 - Compare the degrees of severity between the CES and SVE scales.

- Step 3 - Case-by-case classification according to the qualitative/descriptive analysis, based on the signs and symptoms observed during CES and SVE.

The qualitative analysis of the signs observed in the CES and SVE was carried out once the scales did not provide subsidies for the therapeutic planning. The analysis was based on the comparison of the CES and SVE findings according to the criteria presented on Chart 1.

**Chart 1:** Presents the qualitative correlation criteria of the signs observed at CES and SVE.

Swallowing phases	Clinical evaluation	SVE
		- Anterior deflection
Oral preparatory	Anterior deflection	- Posterior deflection
		- Stasis in the vallecula
	Inefficient chewing	- unchewed food
		- Anterior deflection
	Anterior deflection	- Posterior deflection
		- Stasis in the vallecula
Oral		- Posterior deflection
	- Slow oral transit	- Stasis in the vallecula
		- Oral cavity stasis
		- Stasis in the vallecula
	-Nasal reflux	- Nasal reflux
		- Stasis in the vallecula
		- Stasis in the pyriform sinuses
		- Stasis in the posterior pharyngeal wall
		- Stasis in the upper esophageal sphincter
	- Insufficient laryngeal elevation	- Stasis in the posterior pharyngeal wall
Pharyngeal	- Food returning to the oral cavity	- Stasis in the upper esophageal sphincter
	- Changes to vocal quality	- Penetration/Aspiration
	- Throat secretion	- Penetration/Aspiration
	- Cough	- Penetration/Aspiration
	- Altered auscultation	- Penetration/Aspiration
	- Respiratory frequency alteration	- Aspiration
	- Facial color alteration	- Aspiration

The signs listed above make up the assessment protocol presented in the present study - which is descriptive, providing a broad view of the swallowing process and creation of specific treatment plans, since it is possible to identify the alteration shown.

Each case had its two assessments (CES and SVE) compared according to the qualitative analysis criteria (Chart 1), and was classified in three groups: agreeing (Group 1); disagreeing with clinical evaluation - indicating greater severity (Group 2) and SVE indicating greater severity (Group 3), as shown in Chart 2.

**Chart 2:** Comparison of the qualitative analysis extracted from CES and SVE.

CRITERIUM	DESCRIPTION	Group
Agreeing	ACD e VED apontando mesma severidade	A
Disagreeing	CES indicating greater severity	B
	SVE indicating greater severity	C

- Step 4: Comparing the severity degree classification and the qualitative analysis.

In order to understand the contribution of each procedure in the evaluation of swallowing we chose to analyze the agreement between the qualitative analysis and the degree of severity, by correlating the scales with the data which were identified in the procedures. After the correlation the data were grouped in the following way:

1 - Group in which the CES and SVE severity scale indicated the same degree and the qualitative analysis indicated similar CES and SVE signs;

2 - Group in which the severity scales indicated a greater degree by SVE, correlated with cases in which the qualitative analysis indicated a higher SVE degree, correlated with cases in which the qualitative SVE analysis indicated more signs; 3 - cases in which the CES indicated a greater severity in the scale and more data in the qualitative analysis.

### Statistical methodology

In order to describe the sample profile according to the study variables, we created frequency tables of the category variables (disease) with absolute (n) and percentage (%) values, and descriptive statistics with position and scatter values (mean, standard deviation, minimum and maximum values) of the continuous variable (disease).

The statistical analysis used the “The SAS System for Windows” (Statistical Analysis System), version 8.02 software. The agreement analysis among the classifications used the kappa agreement coefficient. Kappa values above 0.75 meant excellent agreement and values between 0.40 and 0.75 meant intermediate agreement, values below 0.40 indicated low agreement among the classifications. The level of significance adopted for the statistical tests was 5% (p<0.05)^17^, ^18^.

## RESULTS

We studied 30 adult dysphagic patients, 19 men and 11 women. Their mean age was 56 years, varying between 19 and 91 years. They presented different base diagnosis: Parkinson's disease, amyotrophic lateral sclerosis, Machado-Joseph disease, stroke, and four patients with other diagnoses. Chart 3 shows the results from steps 1 and 3.

**Chart 3:** Sample characterization according to age, neurologic diagnosis, dysphagia severity degree in CES and SVE and qualitative analysis criterion.

SUBJECT	AGE	NEUROLOGIC DIAGNOSIS	DEGREE OF SEVERITY		QUALITATIVE ANALYSIS
			ACD	VED	
1	59 a	ELA	2	2	A
2	19 a	AVE	1	2	C
3	39 a	ELA	1	2	C
4	61 a	DP	2	1	B
5	55 a	ELA	2	2	A
6	31 a	ELA	0	1	C
7	37 a	ELA	0	1	C
8	91 a	Presbyphagia	2	2	A
9	48 a	EM	2	2	A
10	75 a	DP	1	2	C
11	56 a	AVE	1	1	A
12	55 a	DMJ	2	0	B
13	60 a	Unclear	2	1	B
14	52 a	DP	2	2	A
15	55 a	AVE	3	3	A
16	45 a	DP	0	0	A
17	46 a	Post-op skull base cyst	1	3	C
18	65 a	DP	2	1	B
19	76 a	DP	1	2	C
20	64 a	ELA	2	1	B
21	46 a	DMJ	1	1	A
22	72 a	DP	1	1	A
23	72 a	DP	1	1	A
24	55 a	DMJ	2	2	A
25	64 a	DP	0	3	C
26	53 a	ELA	0	0	A
27	71 a	DP	1	1	A
28	44 a	DP	0	0	A
29	60 a	DMJ	0	0	A
30	52 a	AVE	2	2	A

Legend: AVE - Stroke

DP - Parkinson's disease

ELA - Amyotrophic lateral sclerosis

DMJ - Machado-Joseph Disease

EM - Multiple Sclerosis

**Graph 1:**
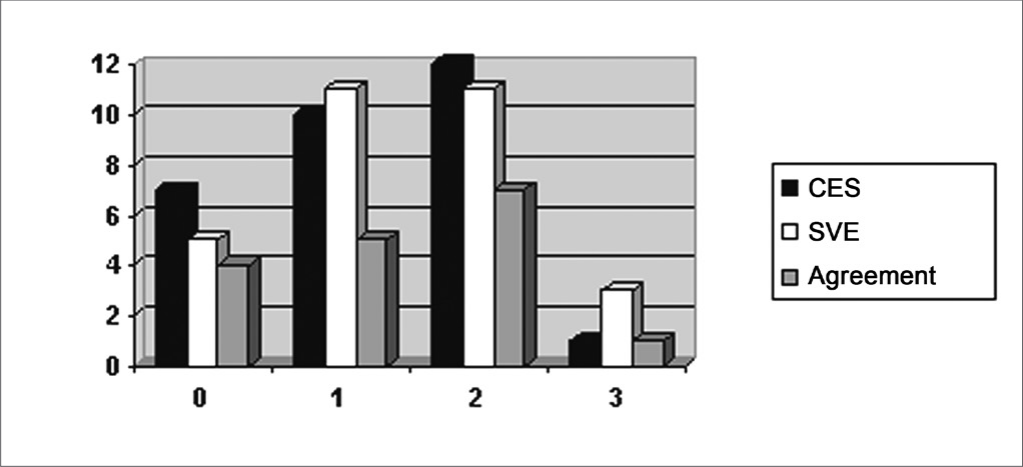
Classification and agreement among the CES and SVE severity scales - CES - Clinical Evaluation of Swallowing SVE - Swallowing Videoendoscopy Evaluation Agreement

The correlation among the results from the severity scale classification in each evaluation procedure (step 2) is depicted on Graph 1.

Graph 1

KAPA=0.400; CI95%: (0.114; 0.686); Z=2.74; p=0.006

It is possible to see in Graph 1 that the number of agreeing evaluations according to the criteria on the severity scales (same degree of severity in CES and SVE) is not very relevant. The statistical analysis indicated an intermediate/low agreement (KAPA = 0.4) in a significant way (p=0.006). Kappa values below 0.4 indicate low agreement.

**Graph 2:**
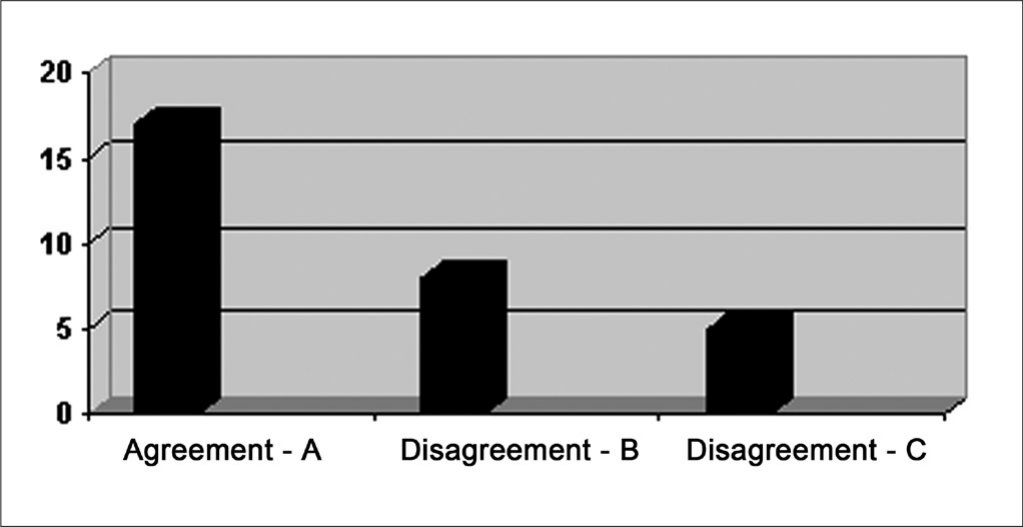
Results from the qualitative/descriptive analysis following the criteria listed in Chart 2. - A - CES and SVE agreeing B - CES points to a greater severity C - SVE points to a greater severity

The results from steps 3 and 4 - descriptive qualitative analysis classification and comparison of these results with the severity degree classification - indicated an agreement in most of the cases studied, as shown on Graph 2. The correlation was carried out by the criteria 1, 2 and 3 aforementioned.

The agreement between the degree of severity and the qualitative analysis indicated excellent degree (Kappa=0.962) in a statistically significant way (P<0.001). Such data reinforces that the qualitative/descriptive analysis proved to be an efficient evaluation method.

## DISCUSSION

The subjects in the sample have ages above 50 years, and this can justify the fact that they frequently present neurodegenerative alterations. There is a prevalence of patients with Parkinson's disease and ALS, followed by MJD and Stroke, for being the main population in the ward.

According to Table 1, the agreement observed between CES and SVE severity scales was intermediate/low in a statistically significant way. Many studies correlate data found in the CES with findings from objective exams. Mathers-Schmidt and Kurlinski^19^ questioned professionals on the criteria used in the clinical assessment and frequency of patient referral for objective evaluation. 78.3% of the physicians who participated in the survey “always” refer the patients to an instrumental assessment, which reinforces the need to perform objective tests.

The correlation of signs observed between CES and SVE, from the qualitative viewpoint is justified by the influence of the oral phase in the pharyngeal phase of swallowing^20^. The presence of deflections is one example. The mobility of the facial muscles is fundamental in the pressurization of the food bolus for its proper ejection^21^, any inadequacy in the muscles causes deflections (anterior - food deflection to the mouth; and posterior - food deflection towards the laryngeal region) and anomalous organization/ejection, altering the swallowing process in the pharyngeal phase. Thus, lip sealing alterations and reduction in the intra-oral pressure can generate posterior deflection or stasis in the vallecula, and these situations can be seen at the SVE. This terminology: “posterior deflection” was used because it states that the swallowing process uses the intraoral pressure action to start the pharyngeal swallowing, in other words, any alteration to this pressure causes the presence of food in the pharynx before the pharyngeal phase starts.

Nasal food reflux, which cause is inefficiency or failure of the soft palate muscles, reducing intraoral pressure - which can be seen at CES and SVE. There are correlations between aspiration risk and changes in soft palate movement^22^.

Motor or sensitive involvement of facial muscles causes alterations to the neuronal information, which is the basis for a proper motor response regarding the food to be ingested. Such involvement may cause an increase in oral transit time, posterior deflection and/or inefficient ejection, resulting in alterations of the pharyngeal phase, especially stasis in the valleculas^23^, seen during SVE.

Multiple swallowing can be seen during CES and in SVE, which happen because of stasis in the oral cavity and vallecula - an inadequate oral phase which causes altered food pushing. Stasis in the pyriform sinuses and posterior pharyngeal walls are also considered alterations to the pharyngeal phase of swallowing, having seen that the mechanism impaired is pharyngeal wall mobility.

Throat clearing, cough and alterations in neck auscultation are signs of penetration and/or aspiration seen during CES and can be confirmed by SVE -by the presence of food in the larynx without passing through the vocal folds (penetration) or by the presence of food below the vocal folds (aspiration)^24^, ^25^ which alters the respiratory frequency and the facial color observed during CES^12^.

International studies comparing the use of neck auscultation, CES and objective exams to predict aspiration indicate that the association of two procedures is safer to find aspiration since the auscultation alone does not guarantee sensitivity to all the altered cases^26^, ^27^, ^28^.

Tohara et al.^29^ suggest the use of three non-objective evaluation tests (water, pudim and x-ray) in order to detect aspiration. The authors point out that the combination of tests carries 90% sensitivity and 71% specificity when compared to VFE and suggest its use for screening purposes. In the present study we observed an excellent agreement - statistically significant between the qualitative/descriptive analysis and the classification of severity scales for the sample (Table 4). The classification by means of qualitative criteria proved to be necessary since the severity scales have a long interval, that is, within the same degree they present different alterations which require specific treatment approaches. As we consider each specific clinical sigh seen at CES and SVE, it is possible to improve care to the population with dysphagia.

As we observe the data individually, we stress 13 disagreeing cases (Graph 2). In 5 of them the clinical evaluation showed more data and in 8 the SVE brought more information. In the CES 5 disagreeing cases presented episodes of cough (2 subjects) and throat clearing (3 subjects), suggesting laryngotracheal penetration/aspiration. In one of the cases, the CES showed throat clearing and the SVE showed stasis in the pyriform sinuses. We can argue that the laryngeal sensitive control and that of the laryngopharyngeal region is the responsibility of the vagus nerve - superior laryngeal branch^13^ which would point to the presence of stasis in the laryngopharyngeal region can also trigger nerve endings, causing throat clearing or cough as motor reactions, leading to a misinterpretation of the laryngeal penetration by the clinical evaluation.

We can argue that in the 8 cases in which SVE showed more data, in two patients we observed stasis which were not suggested in the clinical evaluation, besides aspiration in two and laryngotracheal penetration in four cases.

It is important to discuss the clinical evaluation that is not efficient to identify silent penetrations and aspirations, besides being little efficient to detect stasis in difficult places, which can cause late aspirations and inefficient treatment.

Of the 30 patients investigated, in 26 who were clinically and vide-ofluoroendoscopically evaluated we noticed that it was not safe to forecast the presence of penetration/aspiration of liquids by the clinical evaluation^30^. Furkim et al.^31^ evaluated 32 children by means of CES and VFE. The authors reported that in most cases, suggestive signs of aspiration observed in the clinical evaluation were confirmed by the video-fluoroendoscopy examination, confirming the statement that the objective exam and the CES are complementary to each other in the assessment of swallowing. 30 children had suggestive signs of aspiration in the clinical evaluation; in five (15.6%) aspiration was not confirmed during VFE and in two cases (6.3%) the clinical evaluation did not show suggestive signs of aspiration, though aspiration was seen at the VFE.

Many studies stress the importance of associating the CES and the objective examination in the assessment of swallowing. Such studies suggest that the two procedures are complementary and essential for the diagnosis and treatment planning of dysphagia, leading to the definition of more specific approaches for each patient^33^, ^34^. Because of the need for complementary tests, the present study also presents the evaluation roadmap used in order to suggest assessment procedures which may help to better understand the swallowing process and provide complementary information to the clinical-therapeutic rationale of dysphagic patients.

Swallowing evaluation procedures must try to understand the swallowing process, in other words, the mental cognitive-status behavior^4^, the oral and pharyngeal phases, in order to help in treatment decision. CES and SVE must be complementary and the results of the present investigation reinforce the need to perform an objective test, considering SVE as a doable procedure.

SVE is a highly efficient procedure since it does not require high investments because the equipment utilized is the one ENTs are already used to having, and also in terms of time because the entire test can be performed well under 20 minutes. The SVE also broadens the action scope of otorhinolaryngologists and allows for an interdisciplinary work with speech therapists.

## CONCLUSION

1. The severity classification agreement of CES and SVE proved to be intermediate/low, reinforcing the need to perform both assessment procedures.
2. The agreement between the correlation of severity degree and qualitative/descriptive analysis severity proved to be excellent, reinforcing the qualitative/descriptive analysis as an efficient assessment method.

On-going PhD thesis at the Institute of Language Studies in the field of Neurolinguistics, entitled: “Study and speech therapy follow up of post-stroke subjects”. Such study is associated to the Neurolinguistics Integrated Project (CNPq: 521773/95-4).

**ATTACHMENT 1**

Evaluation Roadmap

***Swallowing Clinical and Video-endoscopic (SVE) Assessment Protocol*** (May/07)

Exam date: _____ / _____ / _____

Patient: ____________________ Reg.#: ____________________ BD: _______________ Age: _____

Address: _________________________________________________________________

Tel: _________________________ Informer: ___________________________________

D.H.: ______________________________________________________________________

P.H: ______________________________________________________________________

Disease Duration: ____________________________________________________________

Medicamentions: ____________________________________________________________

Current complaint: ____________________________________________________________

Swallowing complaint: ()Yes_____()No

Complaint duration: ____________________________________________________________

Prior feeding habits: _______________________________________________________

Meal Records (24 hrs): __________________________________________________

Usual Weight: __________ Current weight: _______________ Height:__________ BMI:

Monthly family income: _________________________ # of family members: __________

General health status:

Heart disorders: ______________________________________________________________________

High blood pressure: ______________________________________________________________________

Pulmonary infections: ____________________________________________________________

Gastric disorders: _________________________________________________________________

Mouth and teeth alterations: ____________________________________________________________

Malnutrition: ______________________________________________________________________

Dehydration: ______________________________________________________________________

Diabetes: (Type) ____________________________________________________________

Tracheostomy: ( ) Present ( ) absent

Cannula: ( ) metal ( ) PVC plastic ( ) silicone ( ) with Cuff ( ) WO/cuff

Mechanical ventilation ( ) Present ( ) absent

Non-invasive ventilation: Mask ( ) Nasal ( )

Swallowing complaint:

**Table:** 

SWALLOWING PHASES							
PRELIMINARY PHASE							
Pleasure eating	Y N						
Appetite	Y N						
ORAL PHASE		Freq	Duration	PHARYNGEAL PHASE		Freq	Duration
Food escapes the mouth	Y N			Nasal reflux	Y N		
Difficulties chewing	Y N			Cough	Y N		
Food stuck to the top of the mouth	Y N			Gagging	Y N		
Difficulties to push food	Y N			Throat clearing	Y N		
Food remains in the oral cavity	Y N			A feeling of stuck food	Y N		
Liquid / saliva deflects from the mouth	Y N			Difficulties swallowing	Y N		
Oral cavity pain	Y N			Swallowing pain	Y N		
Pain/difficulty to swallow saliva	Y N			Longer swallowing time	Y N		

Indirect clinical evaluation

**Table:** 

Face	Mandible
Mobility (VII): ( ) pres ( ) abs ( ) red	Mobility (V): ( ) pres ( ) abs ( ) red
Sensitivity (V): ( ) pres ( ) abs ( ) red	Sensitivity (V): ( ) pres ( ) abs ( ) red
	Biting reflex (V): ( ) pres ( ) abs ( ) red
Lips	Tongue
Mobility (VII): ( ) pres ( ) abs ( ) red	Mobility (XII): ( ) pres ( ) abs ( ) red
Sensitivity (V): ( ) pres ( ) abs ( ) red	Sensitivity (V, IX): ( ) pres ( ) abs ( ) red
	Gustation (VII, IX): ( )pres ( ) abs ( ) red
Oropharynx	Larynx
Mobility (X): ( ) pres ( ) abs ( ) red	Mobility (X): ( ) nl ( ) alt ( ) red
Hyper nasal voice (X): ( ) pres ( ) abs	Voice /a/: G R B A S I -
Sensitivity (IX, V): ( ) pres ( ) abs ( ) red	Voice (Speech): G R B A S I
Vomit reflex - tongue (IX): ( ) pres ( ) abs	Wet voice: ( ) pres ( ) abs
- palate (V): ( ) pres ( ) abs	Laryngeal elevation (X, IX): ( ) pres ( ) abs ( ) red
Gutzman test: ( ) pres ( ) abs	Cough reflex (X): ( ) pres ( ) abs
	Cough (X): ( ) Efficient ( ) Inefficient
Laryngeal auscultation ( ) nl ( ) alt	

Direct clinical evaluation

**Table:** 

Consistency															
		Liquid				Nectar				Honey			Paste		Solid
	3	5	10	C	3	5	10	C	3	5	10	C	sob	sob	
Chewing															E IN
TOL															
Number of swallowing															
Anterior deflection															
Posterior deflection															
Food remains															
Nasal reflux															
Voluntary cough															
Reflex cough															
Cough before															
Cough during															
Cough after															
Wet voice															
Red laryngeal elevation															
Laryngeal auscultation															

Legend: P = present; A = absent; nl = normal; alt = altered

**Signs of penetration/aspiration:**

Facial color alteration: ____________________________________________________

Respiratory rate alteration: ____________________________________________________

O_2_ saturation alteration: ____________________________________________________

Maneuvers utilized: ____________________________________________________

**Swallowing Videoendoscopic Evaluation**

1. Nasal cavities
  Septum ( ) centered ( ) deviated R ( ) deviated L ( ) Non-obstructive irregularities
  Mucosa ( ) pale ( ) edematous ( ) wet ( ) atrophic
  Turbinates ( ) normotrophic ( ) hypertrophic
2. Rhinopharynx:
  Mucosa ( ) pale ( ) edematous ( ) wet ( ) atrophic
  Eustachian tube ostium ( ) free ( ) obstructed
3. Pharynx-soft palate sphincter:
  Phonation
  ( ) Complete closure ( ) Incomplete closure
  ( ) coronal ( ) sagittal ( ) circular ( ) circular with Passavant ring
  Swallowing
  ( ) Complete closure ( ) incomplete closure
  ( ) coronal ( ) sagittal ( ) circular ( ) circular with Passavant ring
4. Hypopharynx (IX, X, XII)
  Tongue base mobility ( ) proper ( ) altered _______________________
  Posterior wall mobility ( ) proper ( ) altered ______________________
  Vallecula ( ) normal ( ) lesion ( ) saliva stasis
  Epiglottis ( ) normal ( ) omega-shape ( ) lesion ______________________
  Arytenoids ( ) normal ( ) hyperemia ( ) edema Interarytenoid region ( ) normal ( ) hyperemia ( ) edema
  Pyriform recess ( ) free ( ) obstructed ( ) saliva stasis ( ) R ( ) L
  Sensitivity ( ) normal ( ) reduced ( ) absent
5. Larynx
  Vocal folds Ventricular folds
  ( ) mobile ( ) normal
  ( ) paresis ( ) R ( ) L ( ) hyperconstriction ( ) R ( ) L
  ( ) Immobility ( ) R ( ) L Laryngeal asymmetry ( ) yes ( ) no
  ( ) arching ( ) R ( ) L sensitivity to the mechanical stimulus
  ( ) atrophy ( ) R ( ) L epiglottis ( ) normal ( ) altered
  ( ) lesion _________________ ( ) R ( ) L aryepiglottic fold ( ) normal ( ) altered
  ( ) other ______________________ subglottis ( ) normal ( ) altered
6. Glottic closure
  ( ) complete ( ) incomplete ( ) consistent ( ) inconsistent
  ( ) posterior triangular slit ( ) mid-posterior triangular slit ( ) anterior spindle-like slit
  ( ) spindle-slit in all the extension ( ) hourglass-like slit

**Table:** 

Classification	Degree	ACD scale (Furkim and Silva,1999)	SVE Scale (Macedo Filho et al., 2000)
Normal	0	No alterations in the oral and pharyngeal phases	No alteration
Mild	1	Difficulties in the oral transportation of the food bolus without signs of laryngeal penetration	Post-swallowing stasis, less than three attempts at clearing, no nasal regurgitation and laryngeal penetration
Moderate	2	Difficulties in the oral transportation of the food bolus, suggestive signs of laryngeal penetration, risk of aspiration and nutritional deficit	Moderate saliva stasis, greater post-swallowing stasis, more than three attempts to push the bolus, nasal regurgitation, reduced laryngeal sensitivity with penetration, no laryngotracheal aspiration
Severe	3	Suggestive signs of laryngeal penetration and aspiration, repetition pneumonias and alterations in the pleasure of eating.	Major saliva stasis, marked worsening of post-swallowing residues, weak or no food pushing, nasal regurgitation, tracheal aspiration.

Swallowing Videoendoscopic Evaluation (SVE)

**Figure:**